# Genome Sequence of Actinobacteriophage LilTerminator, Isolated From *Microbacterium foliorum* on Jekyll Island, Georgia

**DOI:** 10.17912/micropub.biology.001888

**Published:** 2026-01-05

**Authors:** Kristen Darley, Noah English, John Barron, Ellie Brooks, Kamilah Brown, Annale Claxton, Chrishai Cummings, Piper Davidson, Ally Gibson, Jessi Griffin, Amanda Hixson, Faith Holt, Klaire Murray, Phanice Osundwa, Gracie Pilkinton, Lauren Ray, Mikaya Walker, Holly Nance

**Affiliations:** 1 Natural Sciences, College of Coastal Georgia, Brunswick, GA 31520, USA

## Abstract

Actinobacteriophage LilTerminator was isolated from soil collected from Jekyll Island, Georgia, using *Microbacterium foliorum* NRRL B-24224 as the host. The genome of this phage is 39,963 bp, circularly permuted, and contains 62 predicted genes, including one tRNA gene. Based on gene content similarity of at least 35% to actinobacteriophages, LilTerminator is grouped into subcluster EA5.

**Figure 1. SEM of LilTerminator and Phamerator map of genome:**
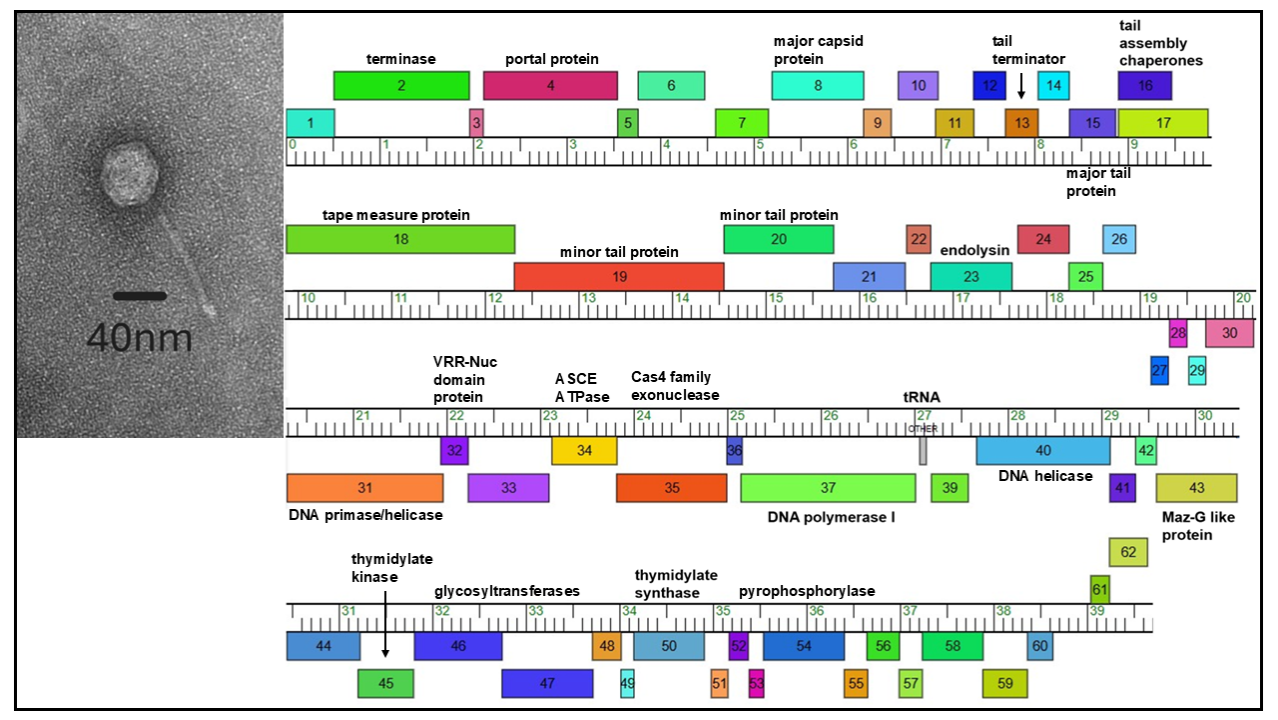
(Left) Transmission electron micrograph of LilTerminator, revealing a siphovirus morphology. Scale bar is 40 nm. (Right) A genome map of LilTerminator. Genes are presented as boxes along a genome ruler (in kilobases). Boxes above the ruler represent genes that are transcribed rightward and those below the ruler leftward, while gene numbers are presented within each box. Different colors reflect different gene families based on sequence similarity. All putative gene functions are labeled.

## Description

The global rise in antiobiotic-resistant bacteria has led to increased focus on bacteriophage, viruses that infect and kill their bacterial host via lysis, as an alternative treatment for infections (Cui et al., 2024). The discovery of new bacteriophages and characterization of their genomes can further increase our understanding of phage biology and their use in medicine. A total of 679 phages isolated using the bacterium *Microbacterium foliorum* as a host have been sequenced and deposited into the Actinobacteriophage database, PhagesDB (Russell and Hatfull 2016). *Microbacterium foliorum* is a rod-shaped, gram-positive bacterium occurring naturally in plant ecosystems but has also been found in wounds and around catheters of immunocompromised patients (Kim et al., 2022). Here, we describe the isolation and characterization of a new actinobacteriophage, LilTerminator, capable of infecting *M. foliorum* .

A soil sample collected from the marshlands of Jekyll Island, Georgia (GPS coordinates: 31.10528 N, 81.40653 W) was incubated in a peptone-yeast-calcium (PYCa) liquid medium at 30°C with shaking for 2 hours. The sample was centrifuged at 2,000 x g for 10 minutes, and the resulting supernatant was filtered through a 0.22 µm pore size. The filtrate was plated with *M. foliorum* and incubated at 30°C on a PYCa plate for 48 hours with soft agar overlay, resulting in the direct isolation of LilTerminator, which produced clear, circular plaques measuring 2-8 mm in diameter (n=15).

LilTerminator was purified through four successive rounds of picking isolated plaques and plating in top agar with *M. foliorum* . A high titer lysate (4.5 x 10 ^9^ pfu/ml) was prepared and used for transmission electron microscopy (FEI Tecnai G2 Spirit BioTWIN) using a 200–400 mesh carbon–formvar-coated copper grid and 1% uranyl acetate for negative staining, revealing that LilTerminator exhibits a siphovirus morphology with a tail length of 187.5 nm +/- 2nm (n=4) and a capsid diameter of 75 nm +/- 2nm (n=4) (Figure 1).

To isolate phage DNA, bacterial DNA/RNA in thelysate was first degraded with a 10-minute 37℃ incubation with DNase I and RNase A, followed by phage capsid denaturation and phage DNA extraction using the Promega Wizard DNA Cleanup System. DNA was sequenced using an Illumina MiSeq 1000 (v3 reagents), with libraries prepped using the NEB Ultra II FS kit. The resulting 150 base raw reads were assembled using Newbler v2.9 (Miller et al., 2010) and genomic termini were assessed using Consed v29 (Gordon and Green, 2013) following Russell (2018). The genome of LilTerminator is 39,963 bp in length with 709-fold coverage. It is circularly permuted and has a G+C content of 63.8%, which is similar to that of its *M. foliorum* host (68.7%).

Initial auto-annotation was conducted using GLIMMER v3.0 (Delcher at al., 2007) and GeneMark v2.5 (Besemer and Boroditsky, 2005). The annotation was refined using Starterator v485 (https://seaphages.org/software/#Starterator), Phamerator (Cresawn et al., 2011) and DNA Master v5.23.6 (Pope and Jacobs-Sera, 2017). HHpred v3.2 searching against the PDB_mmCIF70, SCOPe70, Pfam-A, NCBI_Conserved_Domains databases (Södinget al., 2005), BLASTp searches against the NCBI nonredundant and actinobacteriophage databases (Altschulet al., 1990), and DeepTMHMM v1.0.24 (Hallgren et al., 2022) were used to determine gene functions. Aragorn (Laslett and Canback, 2004) and tRNAscan-SE v2.0 (Lowe and Eddy, 1997) were used to identify tRNA genes. Default settings were used for all software.

LilTerminator has a total of 62 predicted genes, including 61 protein-coding genes, 23 of which have putative functions, and one putative tRNA-Undet (NNN) gene. Approximately half of the genes are transcribed rightward, with the remaining transcribed leftward. LilTerminator was assigned to subcluster EA5 based on gene content similarity of at least 35% to phages in the Actinobacteriophage database, phagesDB (https://phageDB.org) (Russell and Hatfull, 2016; Pope et al., 2017). Like all EA5 phages with annotated genomes, LilTerminator encodes a pair of tail assembly chaperone proteins through a -1 translational frameshift, a DNA polymerase I, a Maz-G like protein and a thymidylate synthase. Also like most other annotated EA5 phages (8 out of 13), LilTerminator encodes an endolysin downstream of the minor tail proteins, although not all the endolysins are grouped into the same protein family. There is no identifiable capsid maturation protease, holin, integrase or immunity repressor function, the latter suggesting this phage is unlikely to establish lysogeny.

**Nucleotide sequence accession numbers**

GenBank Accession Number is PV876929. The Sequence Read Archive (SRA) number is SRX28150555 .
